# How effective are health messages/warnings in improving knowledge and awareness of alcohol-related harm? The Slovenian case on using a mobile app

**DOI:** 10.1186/s12889-023-17353-5

**Published:** 2023-12-11

**Authors:** Sandra Radoš Krnel, Igor Pravst, Maša Hribar, Bojan Blažica, Anita Kušar

**Affiliations:** 1 Analysis and Development Centre, National Institute of Public Health, Trubarjeva Cesta 2, Ljubljana, Slovenia; 2 Nutrition and Public Health Research Group, Nutrition Institute, Koprska Ulica 98, Ljubljana, Slovenia; 3 Biotechnical Faculty, University of Ljubljana, Jamnikarjeva 101, Ljubljana, Slovenia; 4 VIST – Faculty of Applied Sciences, Gerbičeva Ulica 53, Ljubljana, Slovenia; 5 Computer Systems Department, Jožef Stefan Institute, Jamova Cesta 39, Ljubljana, Slovenia

**Keywords:** Alcohol, Health warnings, Alcohol labelling, Mobile app, Alcohol-related harm, Awareness

## Abstract

**Background:**

Consumers generally lack access to information on alcoholic beverages, in spite of it being readily available for food and non-alcoholic beverages. Given the rights of consumers, and as with other products harmful to the population, there have been increasing calls for health warnings to be placed on alcoholic beverages, similar to those implemented on tobacco products. The aim of our research was to assess whether knowledge and awareness of the risks and harms associated with alcohol can be improved with a mobile app.

**Methods:**

Intervention was conducted using VKJ mobile app, which enables users to scan the barcode of an alcoholic beverage and receive feedback on its labelled alcohol content and estimated energy value. At each search, eleven different health messages/warnings about the risks and harms of alcohol are also displayed randomly, rotating on the screen. A survey was conducted before and after the intervention, to assess the knowledge and awareness of the risks and harms associated with drinking alcohol.

**Results:**

Significant differences were found for eight of the twelve tested statements. The improvement was seen to a greater extent in the group of high-risk drinkers. The results also showed that the vast majority of participants (78%) who were exposed to the health messages supported mandatory labelling of alcoholic beverages with information on ingredient listing and energy value, and 72% would like to have health warnings on alcohol products.

**Conclusions:**

The use of a mobile app can be an option to improve knowledge and raise awareness of the risks and harms related to alcohol.

**Supplementary Information:**

The online version contains supplementary material available at 10.1186/s12889-023-17353-5.

## Background

In Slovenia, alcohol drinking is a serious public health problem, both among adolescents and adults. The registered per capita consumption of pure alcohol in Slovenia is above the EU average, as is the mortality rate attributed to alcohol-related causes of death [1, 2]. To achieve better results in reducing alcohol consumption and alcohol-related harm, a comprehensive alcohol policy is needed, incorporating proven and effective measures in different areas of action [3–5]. Alcohol products labelling and health warnings are part of a comprehensive public health strategy to reduce alcohol-related harm. Adding health warning labels (HWL) to alcohol containers is an important first step in raising awareness; in the long term such an approach can support the establishment of greater social understanding of the harmful use of alcohol [6].

Alcoholic beverages are remarkable as consumer products with relatively little consumer information on the label [7]. According to EU Regulation No. 1169/2011, on the provision of food information to consumers, the labelling of ingredients and nutritional declaration is not mandatory for alcoholic beverages with more than 1.2% alcohol by volume; only alcoholic strength by volume is mandatory information on such products [8]. Consequently, consumers generally lack access to information on alcoholic beverages similar to that on food and non-alcoholic beverages [9]. In the World Health Organisation (WHO) Health Evidence Network Synthesis Report the authors highlighted that, in the WHO European Region, labelling regulations were more likely to mandate the listing of ingredients (40% of Member States) than information on nutritional values (19% of Member States; all of which included ingredient listing). In terms of health information labelling, 28% of the WHO European Region Member States have introduced legislation to include some form of health-related information on the label, but Slovenia is not among these countries [10]. Given the rights of consumers, and as done with other products harmful to the population, the messages should clearly reflect the risks of alcohol consumption and provide warnings about its harms to all consumers. There have therefore been increasing calls for health warnings to be placed on alcoholic beverages, similar to those implemented on tobacco products [7, 11]. The importance of the appropriate information on alcoholic beverages is emphasised in the Communication from the Commission to the European Parliament and the Council, on Europe's Beating Cancer Plan; “…the Commission will propose a mandatory indication of the list of ingredients and the nutrition declaration on alcoholic beverage labels before the end of 2022 and of health warnings on labels before the end of 2023”[12].

In recent decades, there has been rapid development of new technologies, including specific tools such as mobile apps, which open up new opportunities for delivering health information and behaviour change interventions. Mobile technology is widely accessible, has the capacity to reach a broad audience, and can be easily and affordably downloaded [13–15]. All these features make mobile apps a unique tool that can be used in public health, and evidence of their effectiveness is indispensable for decisions about their further use. However, despite the large number of mobile apps targeting weight management [16], apps for improving awareness about alcohol-related harm are relatively scarce. Therefore, our research provides some new information about the usefulness of such tools.

In 2019 a smartphone application called “VešKajJeš” (VKJ; English translation: “Know what you eat”) was released in Slovenia [17], within the frame of the Slovenian programme “VešKajJeš” lead by Nutrition institute, supported by the Ministry of Health of Slovenia. The VKJ app enables users to scan the barcode of a selected food item and receive feedback on its nutritional profile. The app has about 35.000 users and was originally developed only for foods and non-alcoholic beverages, but analyses of user behaviour highlighted that more than 30% of searches in the app were targeting alcoholic beverages (which originally were not covered). This could be explained by the fact that app users were seeking additional information about the ingredients of alcoholic beverages, which cannot be found on product labels. Therefore, the VKJ app was upgraded in 2021 with an additional module for alcoholic beverages, which provides information on labelled alcoholic content and estimated energy value. Moreover, this module also presented a new communication channel, enabling awareness to be raised about alcohol-related harm. After being upgraded, the mobile application also enabled the presentation of health messages/warnings about the risks and harms of alcohol, which were developed using Eurocare’s Overview of the Library of Alcohol Health Warning Labels [18].

The aim of our research was to investigate if knowledge and awareness of the risks and harms associated with drinking alcohol can be improved with the upgraded VKJ app.

## Methods

First step was upgrading the VKJ app with an additional module for alcoholic beverages, in which we added health messages/warnings about the risks and harms of alcohol. And second, we investigated if the exposure to these health messages/warnings can be associated with the differences in knowledge and awareness of the risks and harms associated with drinking alcohol.

### Intervention with mobile app VKJ

Intervention was conducted through smartphone application VKJ [17], which is used in Slovenia to support healthy food choices. App is working on mobile phones with Android or iOS platforms, which present vast majority of mobile devices in Slovenia. It is freely available on Google Play and Apple App Store and promoted by governmental and non-governmental organisations, with particular attention for privacy policy. The app does not collect any personal data from users or individual data about scanned foods and drinks.

**Fig. 1:**
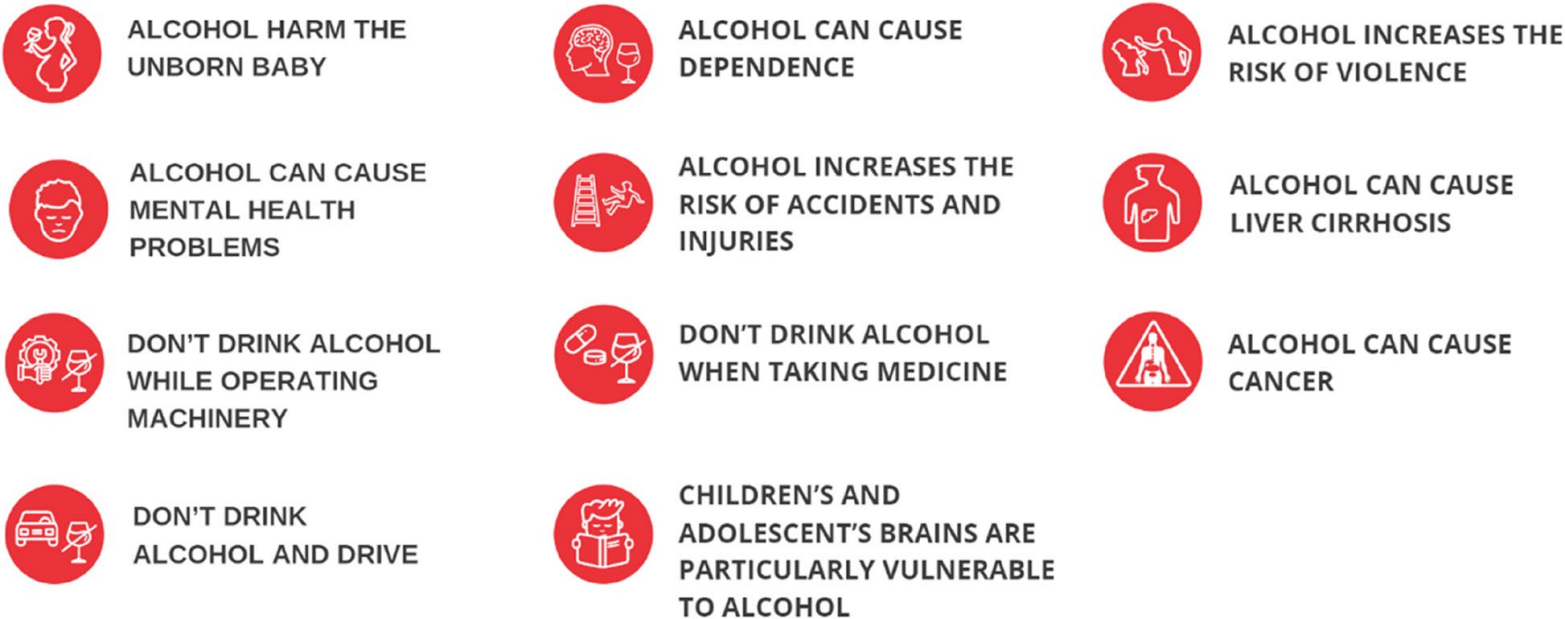
Health messages/warnings about risks and harms from alcohol used in the VKJ mobile app

The upgrade of the VKJ app introduced a module for alcoholic beverages in year 2021, providing information on labelled alcoholic content, estimated energy value and presentation of health messages/warnings about the risks and harms of alcohol; namely eleven different messages are displayed randomly, rotating on the screen (Fig. 1) at each search for an alcoholic beverage. In addition, the Slovenian guidelines for lower-risk alcohol consumption (see Additional file 1) are also presented on the screen (including the message that “the less the better, but the safest is 0 alcohol”), and the app displays a link to a screening tool for assessing personal alcohol consumption (AUDIT-C) with further information on where to get help to reduce drinking [19, 20]. In this way our intervention included three out of the five identified elements that could be useful to consumers: a list of ingredients, nutritional information (energy value), serving size and servings per container, a definition of moderate intake (low risk drinking guidelines) and a health warning label (HWL), as proposed in the review by Martin-Moreno and co-workers [21].

The 2021 upgrade of the VKJ app also introduced option for use mobile push notification with active web links, which was used to disseminate after-intervention study survey among app users.

### The survey

In order to assess if the knowledge and awareness of users of the upgraded VKJ app of the risks and harms associated with drinking alcohol, was changed after exposure to the eleven health messages we designed a survey with 12 statements; nine statements were direct transcription of mentioned health messages, one statement “Alcohol slows reaction time” was representing two health messages (“Don’t drink alcohol while operating machinery” and “Don’t drink alcohol and drive”) and two additional statements (“There is a safe level of alcohol consumption” and “Alcohol can cause asthma”), were used as control questions (Table 2). The study participants assessed these statements using a 4-point Likert scale, ranging from “not at all” to “a very high degree” (“I don’t know” answer was also available). While for ten of the statements, a higher score/stronger agreement meant better knowledge of the risks and harms associated with the use of alcohol, this was not the case with the control questions. If we found that the score was increasing after the intervention also in the control statements, similarly to the other statements, we would not be able to confirm better knowledge about the risks and harms associated with the use of alcohol.

Additional questions in the second part of the survey covered demographics (sex, age and education level), experience with the use of the VKJ app, and information about alcohol consumption during the past 12 months, which was assessed using the short version of the Alcohol Use Identification Test (AUDIT-C). Low-risk drinking was determined by an AUDIT-C sum score of ≤ 4 for women and ≤ 5 for men, as validated for Slovenia [22]. At the end of the questionnaire we asked the participants if they would like to have information on the ingredients and energy value, as well as health warnings related to use of alcohol, labelled on the packaging of alcoholic beverages and other food products that contain alcohol.

### Sample and data collection

We carried out two cross-sectional surveys – the first survey was carried out on general population sample in December 2019 before the upgrade of the VKJ app and intervention, and the second survey in March 2022 (eight months after upgrading the mobile VKJ app) specifically on VKJ app users. Study was conducted using on-line questionnaire, programmed with online open-access tool 1KA (University of Ljubljana, Slovenia; URL: https://www.1ka.si/). A multi-channel non-probability convenience sampling approach was used for the first survey. Invitation for participation in the study (with survey link) was published on the channels of project partners (web page, social media). Considering that second survey was launched after the upgrade of VKJ app, we were able to specifically access VKJ app users through push notifications system. All VKJ app users with activated push notification functionality on their mobile phones received invitation for participation in the study (with survey link).

### Statistical data analysis

The analysis was carried out using the SPSS program, Version 27, IBM. Chi-square statistical test was used for comparison of the sociodemographic characteristics of both survey samples (after exclusion of answers ‘I don’t want to answer’ and ‘I don’t know’), except for age, where independent samples T-test was used. Significance level was set at 0.05. Questions related to knowledge dimensions were analysed using the Chi-square statistical test, with prior weighting for response frequency, and values for the 2-sided Fisher’s Exact test. For the purposes of the analysis we combined the answers on the 4-point Likert scale into two groups: “I do not agree” and “I agree”, excluding the response “I do not know”. To calculate the differences in knowledge and awareness of the risks and harms associated with drinking alcohol in the first survey, we used the data from all the respondents (naïve respondents, who were not exposed to the intervention). In the second survey, however, we only used the data from those respondents who were users of the VKJ app, and who were therefore exposed to health messages/warnings about the risks and harms of alcohol. Thus, a quasi-experimental approach was used to assess the possible changes in knowledge and awareness about the risks and harms associated with drinking alcohol.

## Results

For the data analyses we only used fully completed surveys. We excluded incomplete responses in both study samples (N of excluded responses = 715 and 2,350, respectively). In the second study sample we also excluded those who did not specifically report the use of the VKJ app (*N* = 404), because they were not subject to our intervention. The final sample therefore included *N* = 1,082 respondents in the first survey, and *N* = 702 respondents in the second survey. The average time to complete the survey was 2 min 46 s. Comparison of socio-demographic characteristics did not show statistical difference between both samples for sex (*p* = 0.473), but it should be noted that the proportion of women was higher than in the overall Slovenian population (51.1%, data from SURS, 2021); namely 59% (*N* = 644) in the first survey and 58% (*N* = 406) in the second survey. The average age was 39 in the first sample and 49 years in the second one, the statistical difference was confirmed between the samples (*p* < 0.001), Of all the respondents, 73% (*N* = 787) in the first survey and 80% (*N* = 563) in the second were recognised as low-risk drinkers, with average AUDIT-C scores of 3.72 and 3.64 (Table 1). The results showed that the vast majority of participants (78%; *N* = 547) who had been exposed to health messages within the intervention supported mandatory labelling of alcoholic beverages with information on ingredients and energy value, while 72% (*N* = 506) would like to see health warnings on alcohol products. Although we observed a positive perception of ingredient listing and health warning messages before the intervention (69% (*N* = 748) and 57% (*N* = 613) respectively), the difference between both samples reached statistical significance for both questions (*p* < 0.001).

**Table 1:** Socio-demographic characteristics, alcohol-related characteristics and responders’ interest in getting information about alcoholic beverages (both surveys: *N* = 1,082 vs. 702)

Variable	Attribute	N_first_ (%)	N_second_ (%)	*P*-value
Sex	Male	430 (40)	291 (41)	0.473^*^
	Female	644 (59)	406 (58)	
	I don’t want to answer	8 (1)	5 (1)	
Age Mean (SD)		39.4 (13.9)	48.9 (14.7)	< 0.001
Drinking habits	Low risk drinkers	787 (73)	563 (80)	< 0.001
	High risk drinkers	295 (27)	139 (20)	
Would you like to have ingredient listing on labels of alcoholic beverages and food products containing alcohol, including energy values?	Yes	748 (69)	547 (78)	< 0.001^*^
	No	170 (16)	39 (6)	
	I don’t know	164 (15)	116 (16)	
Would you like alcohol health warnings to be presented on the packaging of alcoholic beverages and food products containing alcohol?	Yes	613 (57)	506 (72)	< 0.001^*^
	No	330 (30)	92 (13)	
	I don’t know	139 (13)	104 (15)	

*SD* standard deviation; ^*^“I don’t want to answer” and “I do not know” responses were excluded from statistical comparison

Notable differences in knowledge and awareness of the risks and harms associated with drinking alcohol were found between both samples (Table 2). Statistically significant differences were found for eight of the twelve statements, with p-values ranging from 0.000 to a maximum of 0.020. The two control statements were included in these eight: in one case (“There is a safe level of alcohol consumption”) we found a decrease in the score which corresponded to improving knowledge and awareness of alcohol-related harm; however, in the second case (“Alcohol can cause asthma”) there was an increase in the score. For four statements the difference in the score between the two surveys did not reach a statistical significance. In general, the improvement was seen to a greater extent in the group of high-risk drinkers (AUDIT-C sum score of > 4 for women and > 5 for men; p-values ranged from 0.000 to 0.015), with significant improvement in awareness for six of the statements (“Alcohol increases the risk of violence”; “Alcohol can cause changes in the brains of children and adolescents”; “Alcohol can cause cancer”; “Alcohol can cause dependence”; “Alcohol increases the risk of accidents and injuries”; and “Alcohol can cause asthma”), while for low-risk drinkers there was a significant improvement in only three (“Alcohol increases the risk of violence”; “There is a safe level of alcohol consumption”; and “Alcohol can cause cancer”).

**Table 2:** Knowledge and awareness of the risks and harms associated with drinking alcohol in the two surveys

Survey implementation		First survey	Second survey	*P*-value
		**N (%)**^a^	**N (%)**^a^	
Drinking alcohol during pregnancy may harm the unborn child	I do not agree (including "not at all")	12 (1)	10 (1)	0.662
	I agree (including "to a very high degree")	1050 (99)	683 (99)	
	Total	1062	693	
Alcohol slows reaction time	I do not agree (including "not at all")	17 (2)	4 (1)	0.071
	I agree (including "to a very high degree")	1059 (98)	693 (99)	
	Total	1076	697	
Alcohol can cause liver cirrhosis	I do not agree (including "not at all")	9 (1)	3 (0)	0.385
	I agree (including "to a very high degree")	1060 (99)	687 (100)	
	Total	1069	690	
Alcohol increases the risk of violence	I do not agree (including "not at all")	62 (6)	11 (2)	0.000
	I agree (including "to a very high degree")	1012 (94)	686 (98)	
	Total	1074	697	
Alcohol can cause changes in the brains of children and adolescents	I do not agree (including "not at all")	24 (2)	5 (1)	0.020
	I agree (including "to a very high degree")	1008 (98)	644 (99)	
	Total	1032	649	
Alcohol can cause mental health problems	I do not agree (including "not at all")	43 (4)	14 (2)	0.019
	I agree (including "to a very high degree")	1008 (96)	675 (98)	
	Total	1051	689	
There is a safe level of alcohol consumption	I do not agree (including "not at all")	252 (24)	230 (35)	0.000
	I agree (including "to a very high degree")	782 (76)	436 (65)	
	Total	1034	666	
Alcohol can cause cancer	I do not agree (including "not at all")	162 (21)	61 (13)	0.000
	I agree (including "to a very high degree")	604 (79)	427 (87)	
	Total	766	488	
Alcohol can cause dependence	I do not agree (including "not at all")	26 (2)	5 (1)	0.008
	I agree (including "to a very high degree")	1052 (98)	694 (99)	
	Total	1078	699	
Alcohol increases the risk of accidents and injuries	I do not agree (including "not at all")	25 (2)	4 (1)	0.004
	I agree (including "to a very high degree")	1051 (98)	692 (99)	
	Total	1076	696	
Alcohol can interfere with certain medications	I do not agree (including "not at all")	14 (1)	3 (0)	0.082
	I agree (including "to a very high degree")	1038 (99)	674 (100)	
	Total	1052	677	
Alcohol can cause asthma	I do not agree (including "not at all")	215 (67)	102 (51)	0.000
	I agree (including "to a very high degree")	106 (33)	97 (49)	
	Total	321	199	

^a^“I do not know” responses were excluded for each statement separately, meaning that the number of responders in the analyses is somewhat different in different statements

## Discussion

Our study showed that health warnings presented as written messages together with pictograms can support communication of knowledge of alcohol-related harm and risks, as stated in Babor’s third edition of “Alcohol: No Ordinary Commodity” and in other research papers [23–29]. The users of the VKJ app had a significantly better knowledge of alcohol-related harm and risks after the intervention in six domains, namely “Alcohol increases the risk of violence”, “Alcohol can cause changes in the brains of children and adolescents”, “Alcohol can cause mental health problems”, “Alcohol can cause cancer”, “Alcohol can cause dependence” and “Alcohol increases the risk of accidents and injuries”. The highest difference in awareness was found when communicating “Alcohol can cause cancer”, which was the least known risk among the study participants. In the case of well-known facts such as “Drinking alcohol during pregnancy can harm the unborn child”, “Alcohol slows reaction time”, “Alcohol can cause liver cirrhosis” and “Alcohol can interact with certain medications” the difference between scoring of both study samples did not reach statistical significance, which reflects the fact that the participants already had knowledge about these risks and harms (correct answers in the first survey varied between 98 and 99% of the participants).

In order to avoid acquiescence bias, we used two control questions (“Alcohol can cause asthma” and “There is a safe level of alcohol consumption”) in which lower scoring or stronger disagreement indicated a better knowledge about the risks and harms associated with the use of alcohol. The control question “Alcohol can cause asthma” was not recognised as such, and the percentage of those who agreed with this (incorrect) statement even increased after the intervention; interestingly, about 50% of respondents were not aware that alcohol does not cause asthma. However, in the case of the second control statement, “There is a safe level of alcohol consumption”, we observed better knowledge in app users after the intervention. While more than 75% of the respondents of first survey agreed with the statement, this decreased to 65% among app users after the intervention. This indicates that more than half of our participants were not aware of the fact the alcohol is a toxic, carcinogenic substance which poses a risk at any level of consumption, and the level of consumption that minimises an individual's risk is 0 g of ethanol per week [30–32].

In general, the results of our study are in line with those of some previous studies, showing, for example, that some alcohol-related health messages such as “Drinking alcohol during pregnancy can harm the unborn child”, “Alcohol slows reaction time”, “Alcohol can cause liver cirrhosis” and “Alcohol can cause mental health problems” are more commonly known [33]. Despite a growing body of evidence that the consumption of alcoholic beverages is causally linked to several different cancer types, the study participants were less sure about whether alcohol can cause cancer [33–35], even though the Slovenian correspondents expressed greater awareness. Hobin et al. [25] concluded that in a real-world setting, cancer warning labels are noticed and increase the knowledge that alcohol can cause cancer, and this was also the case in our study. Therefore, adding cancer warnings to alcohol labels, similar to those used on tobacco products, may deter people from purchasing alcohol products and increase awareness of the causal link with cancer, which could then confer increased public support for alcohol policies [35–37]. Other types of interventions have also been shown to be efficient. For example, a study conducted by Christensen et al. [38] showed that a mass media campaign was associated with an increase in awareness of alcohol as a risk factor for cancer [25], as well as with alcohol policy support at a population level. It seems that lesser known alcohol-harm messages have stronger effects.

Our study also revealed interesting results depending on the drinking habits of the study participants. Participants with a higher alcohol consumption (high risk drinkers with an AUDIT-C sum score of > 4 for women and > 5 for men) showed lower awareness of alcohol-related health risks and harms, and a stronger belief that “There is a safe level of alcohol consumption” than participants with a lower AUDIT-C sum score, which has been already reported in other studies [39, 40]. The difference in awareness between these groups in preintervention survey varied greatly in all the observed domains (p values ranged from 0.000 to 0.015). However, in a post-intervention survey, higher change in knowledge was seen in the group of high-risk drinkers. This can be explained by the fact that it is easier to improve and reach statistical significance when baseline knowledge is lower, compared to the situation where the participants already have knowledge about alcohol-related risks and harms before intervention. We observed a significant difference for six statements in the group of high-risk drinkers, while for low-risk drinkers the difference was significant only for three. Similarly, in an Australian study the authors found exposing at-risk drinkers to warning statements related to specific chronic diseases increased the extent to which alcohol was believed to be a risk factor for those diseases, and influenced consumption intentions [41]. With regard to the lower awareness in the high-risk group, some scholars have proposed that these consumers relativize the perceived risk of their (high) consumption to reduce the cognitive dissonance between their behaviour and the risk they expose themselves to [40]. If this is true, we wonder about the reason for the greater improvement in knowledge in this group. If the alcohol health warnings were placed on the product itself, we could argue that high-risk drinkers were more exposed to HW, but in our case users had to use the mobile app to get additional information and to see the HW. This question needs further research to be properly addressed.

Finally, we would like to emphasize that the majority of study participants (69% and 78% in the first and second survey, respectively) supported the introduction of mandatory labelling of ingredients and energy content on alcoholic beverages, which is much higher support than in the results of some international surveys [9, 42]. Thus, the responses of the study participants also provided an important message to policymakers; not just with regard to the introduction of labelling that includes information on ingredients and nutritional information, but also with regard to the introduction of alcohol health warnings highlighting the harm done by alcohol (supported by 57% and 72% of participants, respectively). As stated in a recent scoping review, alcohol health warning labels are an important tool for raising awareness on alcohol-related risks, as part of wider alcohol policy approaches [43].

### Limitations

The present study faced several limitations. Study survey was conducted using two different sampling approaches, because active push notifications were only introduced into the VKJ app together with the upgrade, which was used for the study intervention. The study design was therefore quasi-experimental with two cross sectional samples, one on general population and one specifically on VKJ app users. Considering that the participants in first survey were recruited using convenience sampling, the observations are not transferable to the population level. Furthermore, because the second survey was conducted solely on VKJ app users, we cannot exclude that such population could have somewhat different health-related lifestyle and knowledge, and is more sensitive for health-related messages. A better approach would be to use the same study sample of app users in both surveys, but in our case such an approach was not feasible because the VKJ app is fully anonymous and does not allow the collection of any user data. Therefore, we were unable to link actual app users to the survey responses. Different sampling approach also resulted is some socio-demographic differences between samples. the study samples differed according to the mean age (39 vs. 49), and women were overrepresented in both samples (60% and 58% respectively). The sampling approach we used likely included a somewhat higher proportion of health-oriented participants in comparison to the general population, due to the fact that the invitation for participation was sent using different channels operated by health-oriented organizations and through VKJ app. It should be also mentioned, that the effectiveness of the intervention is most likely correlated with the exposure to the intervention, where frequency of exposure could be tested as potential moderator. However, due to very strict privacy policy of the VKJ app, such data is not collected by the app and was therefore not investigated. Another limitation is, that study was not designed in a way to measure to what extend improved knowledge might translate into the improved behaviours. Future research should also address this topic.

## Conclusions

While we are waiting for the introduction of mandatory information on ingredients and nutritional information, as well as alcohol health warnings, some alternative interventions designed to raise awareness of health risks or to change behaviour can be implemented. The use of a mobile app as in our study is one of the options that can improve consumers’ knowledge of the risks and harms related to alcohol. However, these interventions need to be supported and reinforced by other policies that influence alcohol consumption. And the other way around, awareness of the risks and harms related to alcohol is, in turn, linked to changes in social norms and increases public support for more stringent alcohol policies. Future research needs to explore the long-term effects of repeated exposure to the same message content in influencing not just knowledge, but also drinking behaviour.

### Supplementary Information

**Additional file 1:** **Appendix 1.** Limits for less hazardous alcohol consumption.

## Data Availability

The datasets used and/or analysed during the current study are available from the corresponding author on reasonable request.

## Abbreviations

AUDIT-C: Alcohol Use Identification Test
HWL: Health Warning Labels
SURS: Statistical Office of the Republic of Slovenia
VKJ app: Smartphone application VešKajJeš (English translation: “Know what you eat”)
WHO: Wolrd Health Organisation
