# Supplementary material for: How effective are health messages/warnings in improving knowledge and awareness of alcohol-related harm? The Slovenian case on using a mobile app
Source: BMC Public Health. 2023 Dec 11;23:2467. doi: 10.1186/s12889-023-17353-5 (PMC10712114; doi:10.1186/s12889-023-17353-5)
Supplement: Supplementary file 1 — Additional file 1: Appendix 1. Limits for less hazardous alcohol consumption. [file 12889_2023_17353_MOESM1_ESM.docx]

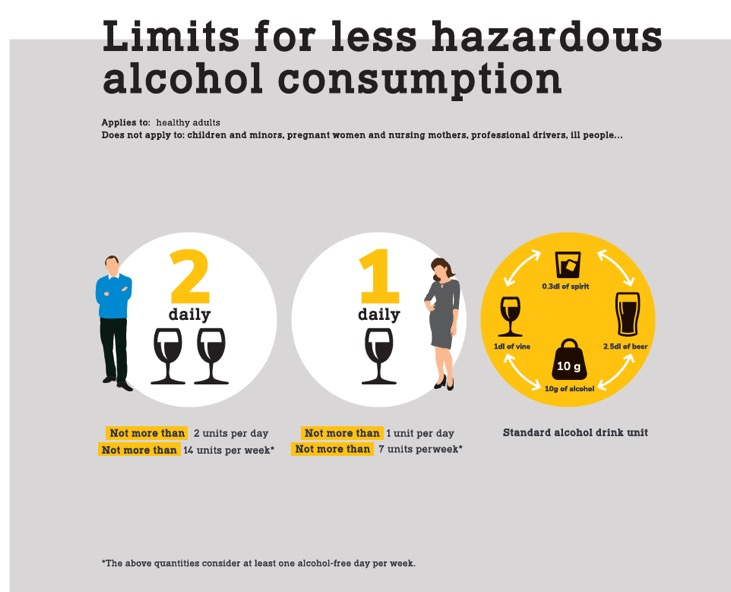


Source: National Institute of Public Health. SOPA Project – Together for a responsible attitude towards alcohol consumption. <https://www.sopa.si/en/alcohol/alcohol-consumption/>
